# Beyond the Normal Ejection Fraction: Can Strain Echocardiography Unmask Early Myocardial Impairment in Adolescents with Substance-Use Disorders

**DOI:** 10.3390/children13101398

**Published:** 2026-10-14

**Authors:** Oguzhan Ay, Ecem İnce Arslan, Enis Sargın, Ayse Simsek

**Affiliations:** 1 Department of Pediatrics, Division of Pediatric Cardiology, Izmir Democracy University Buca Seyfi Demirsoy Teaching and Research Hospital, 35390 Izmir, Turkey; 2 Department of Child and Adolescent Mental Health and Diseases, Izmir Democracy University Buca Seyfi Demirsoy Teaching and Research Hospital, 35390 Izmir, Turkey

**Keywords:** adolescent substance use disorder, speckle-tracking echocardiography, global longitudinal strain, subclinical myocardial dysfunction, cardiotoxicity, early cardiac screening

## Abstract

**Highlights:**

**What are the main findings?**

• Global and segmental longitudinal strains were significantly impaired in adolescents with substance-use disorder.
• GLS discriminated between the patient and control groups with high diagnostic power and remained an independent predictor in multivariable analysis.

**What are the implications of the main findings?**

• Strain echocardiography may reveal subclinical myocardial injury in adolescent with substance-use disorder whose ejection fraction, although modestly reduced, remains within the normal range; this supports the inclusion of GLS as a routine screening parameter for early cardiac assessment in Child and Adolescent Substance Dependence Treatment Center-type surveillance protocols.
• The diffuse pattern of segmental involvement suggests a toxic, autonomic, or metabolic myocardial effect related to polysubstance exposure, and the findings should not be attributed to a single substance. Because of the cross-sectional design of this study and potential confounding by smoking and polysubstance use, reversibility with abstinence and prognostic value can only be clarified through prospective, longitudinal studies.

**Abstract:**

**Background:** Substance use among adolescents is a growing global health problem, and psychoactive substances may damage the myocardium before conventional echocardiographic parameters change. We aimed to determine whether two-dimensional speckle-tracking strain echocardiography can unmask early myocardial impairment in adolescents with substance-use disorder. **Methods:** In this retrospective cross-sectional case–control study, 29 adolescents with substance-use disorder followed at a Child and Adolescent Substance Dependence Treatment Center and 29 age- and sex-matched healthy controls underwent conventional echocardiography and strain analysis (segmental, territorial, and global longitudinal strain [GLS]) performed by two blinded pediatric cardiologists. Between-group comparisons were adjusted for multiple testing; discriminative performance was assessed by receiver operating characteristic analysis with bootstrap internal validation, and multivariable logistic regression tested the independence of the association. **Results:** Patients had a median substance exposure of 3 years, and 75.9% reported polysubstance use (most commonly pregabalin, 41.4%, and methamphetamine, 34.5%). Current cigarette smoking was reported by 75.9% of patients and none of the controls. Ejection fraction was lower in patients but remained within the normal range (62.3% vs. 68.4%; *p* = 0.016). GLS was significantly impaired in the patient group (−17.0% vs. −20.4%; *p* < 0.001; median difference 3.4 percentage points; Hedges’ g = 2.00), with diffuse segmental involvement across all three apical views. GLS discriminated patients from controls with an area under the curve of 0.913; at the Youden-optimal cut-off of −19.3%, sensitivity was 79.3% and specificity 96.6%. **Conclusions:** Strain echocardiography can unmask early myocardial impairment in adolescent substance and tobacco users, while ejection fraction and other conventional parameters, although differing modestly, remained within their normal reference ranges. Adding GLS to routine cardiac surveillance may improve early detection; however, reversibility with abstinence and prognostic value require prospective longitudinal studies.

## 1. Introduction

Substance use has become a serious global public health crisis among adolescents in recent years [1]. Combating substance dependence constitutes a multidimensional and complex community mental health problem for families, healthcare professionals, and policymakers [2,3]. In particular, the use of readily accessible substances such as cannabis, methamphetamine, and synthetic derivatives may lead to both acute cardiac pathologies and unpredictable sudden cardiac events in young populations [4,5].

The acute and chronic effects of substance use on the cardiovascular system have been widely documented in the literature, particularly in relation to mechanisms such as sympathetic nervous system activation, vasospasm, and myocardial ischemia [6]. Substance use is often not limited to a single agent but may involve polysubstance use [3]. This may increase the potential for cardiovascular damage through the combined effects of different toxic agents and may lead to a more complicated clinical presentation [7]. In particular, the concomitant use of different psychoactive substances may accelerate the transition of subclinical dysfunction to clinically apparent disease by more rapidly depleting myocardial reserve [8]. Furthermore, cases of acute coronary syndrome and myocarditis developing after psychoactive substance exposure clearly demonstrate that these toxic agents can cause substantial damage to cardiac reserve [9].

When conventional echocardiographic methods are insufficient to detect subtle changes in myocardial function, strain echocardiography offers the opportunity to quantitatively analyze the regional and global mechanical properties of the myocardium [10]. As a more sensitive indicator of myocardial deformation, this method plays a critical role in the early detection of subclinical myocardial injury—defined here as impairment detectable by deformation imaging while conventional parameters remain within their normal reference ranges [10]. This advanced imaging technique, which is expected to become an integral part of clinical practice in the future, has the potential to detect myocardial deterioration in asymptomatic individuals considerably earlier than standard echocardiographic measurements [10]. Large-scale, long-term cohort studies specifically focused on substance dependence in the pediatric population remain highly limited; most of the available evidence is restricted to case reports or cross-sectional studies involving late-adolescent and young-adult populations [11,12]. This lack of data makes it difficult to fully characterize the true prevalence, pathophysiological mechanisms, and long-term cardiovascular outcomes of substance use-related cardiotoxicity in children and adolescents [5,13].

The primary aim of this study is to detect an early myocardial impairment that may be overlooked during routine clinical evaluations in children and adolescents with substance-use disorder using strain echocardiography. Through the quantitative assessment of myocardial deformation, this study seeks to identify cardiac effects that may emerge before any apparent deterioration in conventional echocardiographic parameters. Regular monitoring of these early findings may contribute to more effective management of cardiac risk and the prevention of progressive myocardial damage. Accordingly, this study aims to reduce substance use-related cardiac morbidity, preserve long-term quality of life, and facilitate the earlier, evidence-based development of monitoring and treatment strategies for children and adolescents.

## 2. Materials and Methods

**Figure 1:**
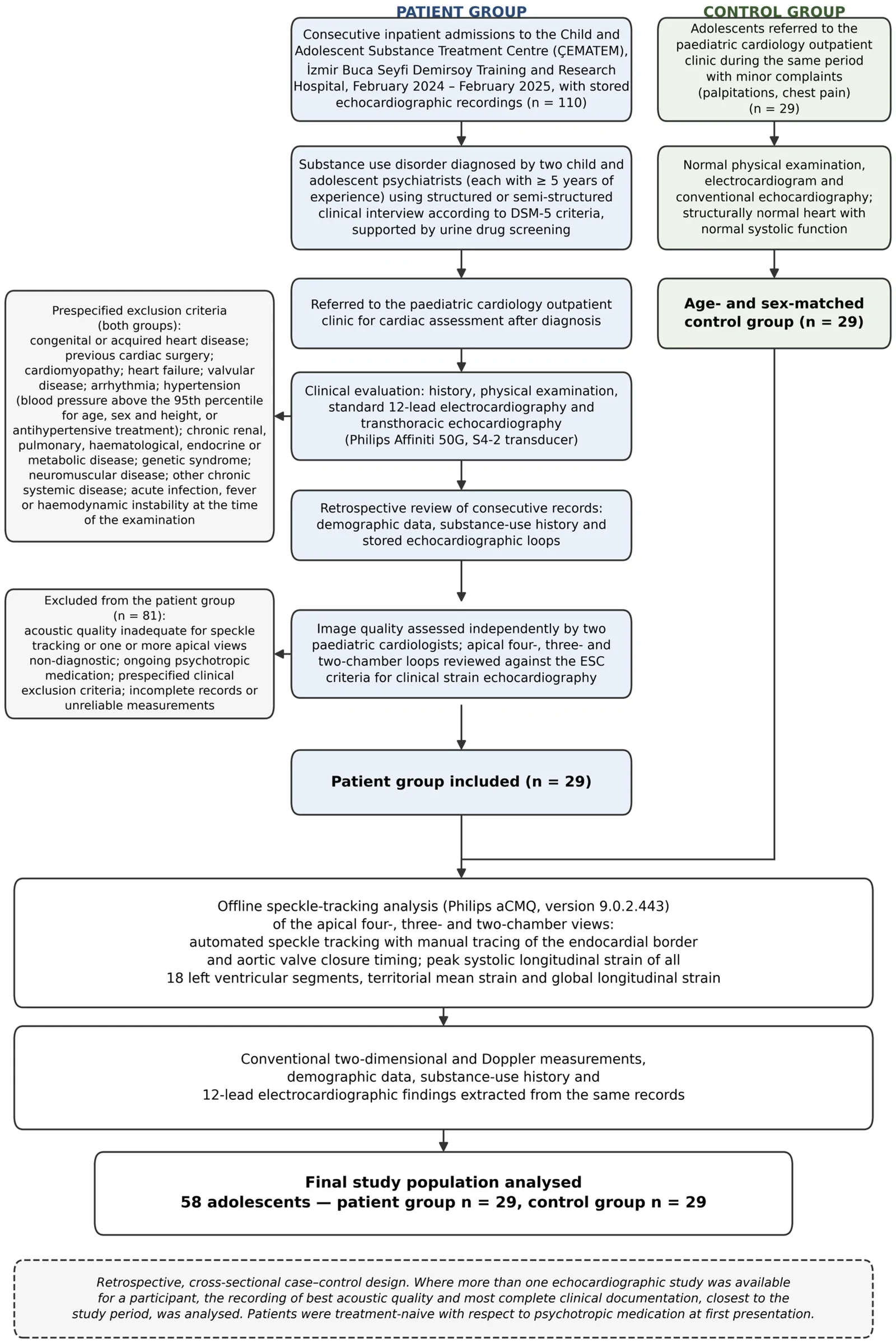
Flow of participants through this study.

This study was designed as a retrospective, cross-sectional case–control study. Following approval by the institutional ethics committee, data collection was initiated, and the study population was formed from patients who presented to the pediatric cardiology outpatient clinic during the last one-year period (February 2024–January 2025). The patient group consisted of 29 adolescents with substance-use disorder who were followed at the İzmir Buca Seyfi Demirsoy Training and Research Hospital Child and Adolescent Substance Dependence Treatment Center (CASDTC)—its official name in Turkey is Cocuk ve Ergen Madde Bağımlılıgı Tedavi Merkezi (CEMATEM)—and referred to the pediatric cardiology outpatient clinic by the child psychiatry unit, along with 29 control participants. All consecutive records containing images of sufficient quality for strain analysis were included; records that did not meet the inclusion and exclusion criteria are detailed in the flowchart (Figure 1). Of the 110 patient records with strain echocardiography data available during the study period, 29 constituted the patient group, and 29 control participants were additionally included. The images were retrospectively retrieved from the echocardiographic image archive stored in the polyclinic record system. The remaining 81 patients were excluded because they met the exclusion criteria, were receiving psychiatric medication, or had inadequate image recordings. The patients included in this study consisted of individuals presenting to CASDTC for the first time who had not received any psychiatric medication. On pediatric cardiology examination, no physical or cardiorespiratory findings were detected, and their physical examinations were normal.

### 2.1. Definition and Operation of CASDTC

Child and Adolescent Substance Use Treatment Centers, also referred to in the literature and official legislation as Child and Adolescent Detoxification Centers or Child and Adolescent Alcohol and Drug Dependence Treatment and Detoxification Centers, are specialized healthcare units serving children and adolescents aged 18 years and younger. These centers provide multidisciplinary services for the medical detoxification, psychiatric evaluation, psychosocial rehabilitation, and long-term follow-up of young people who use alcohol or other substances. The Ministry of Health has published the “Minimum Operating Standards Guide” to establish the operational framework of these centers and ensure the provision of services according to consistent standards [14]. Depending on clinical needs and the severity of the substance-use disorder, CASDTCs offer outpatient or inpatient treatment options. Through early intervention and age-specific, structured treatment programs, they play a critical role in preventing the chronic progression of substance-use disorders [3].

Izmir CASDTC was established in 2012 within a public Training and Research Hospital located in İzmir. It is the only hospital in İzmir providing inpatient treatment for children and adolescents with substance-use disorders. The center provides services through an inpatient unit and two full-time outpatient clinics.

### 2.2. Process of Psychiatric Follow-Up and Treatment Decision-Making for Patients

After children and adolescents who use substances and wish to discontinue their use voluntarily apply to our center for treatment, diagnostic evaluation, differential diagnosis, medical treatment, substitution therapy, and psychosocial treatment processes are initiated. Psychiatric evaluations are conducted in our unit by two specialist physicians in Child and Adolescent Psychiatry, each with at least five years of professional experience. Substance-use disorder is assessed using structured or semi-structured clinical interviews, scales, and laboratory tests guided by the criteria established in diagnostic manuals. Objective evidence of substance use is frequently sought by screening urine samples for substances or their metabolites [15]. Diagnoses are established through clinical interviews based on the fifth edition of the *Diagnostic and Statistical Manual of Mental Disorders* published by the American Psychiatric Association [16]. During treatment, patients’ urine substance-panel analyses and biochemical parameters are monitored periodically, while comorbid psychiatric conditions are systematically assessed and treated using standardized scales. All patients undergoing treatment receive a general pediatric examination; those treated in the inpatient unit additionally undergo screening for potentially transmissible infections [17]. To assess the cardiac risk profiles of patients undergoing treatment in our unit, electrocardiographic and echocardiographic examinations are incorporated into individualized treatment strategies, particularly to minimize potential cardiovascular adverse effects that may develop following psychoactive substance exposure.

Patients presenting to the CASDTC outpatient clinic were assessed according to the DSM-5 criteria to establish a diagnosis of substance-use disorder. An inpatient or outpatient treatment pathway was then determined based on the severity of the patient’s disorder. After diagnosis, patients receiving inpatient treatment were referred to the pediatric cardiology outpatient clinic for cardiac evaluation. A total of 110 patients were admitted to the inpatient unit of our CASDTC; after evaluating those with adequate imaging records according to the exclusion criteria and excluding patients receiving psychiatric medication or meeting other exclusion criteria, 29 patients were found to have imaging records suitable for strain analysis.

### 2.3. Data Collection, Conventional, and Strain Echocardiography Protocol

Demographic data, psychiatric and cardiac examination findings, standard 12-lead electrocardiograms, and recorded echocardiographic images obtained using an S4-2 transducer on a Philips Affiniti 50 G echocardiography system were included for patients followed at CASDTC. Image quality was assessed by two pediatric cardiologists; recordings in which the endocardial borders of all 18 standard segments were visible were considered adequate. The data of patients deemed suitable for strain echocardiographic assessment by the pediatric cardiologists were retrospectively analyzed using the aCMQ strain echocardiography software (Version 9.0.2.443) installed on the Philips echocardiography system, with the aim of determining the correlation of myocardial strain values. Patient data with poor image quality were excluded. A control group consisting of age- and sex-matched healthy individuals was established for the analysis, with the aim of identifying statistically significant differences in left ventricular global strain values associated with substance exposure.

The echocardiographic measurements and strain images included in this study met the criteria specified in the European Society of Cardiology’s *Clinical Applications of Strain Echocardiography* guideline [18]. Studies without adequate image recordings were excluded. Images were analyzed using the Philips Affiniti 50 echocardiography system and the Philips aCMQ strain software used in the Pediatric Cardiology Outpatient Clinic. Analyses were performed using apical four-chamber, apical three-chamber, and apical two-chamber views of the left ventricle. The frame rate, which ranged from 50 to 80 frames per second, was determined automatically by the Philips system on the basis of the ECG signal; the aCMQ software acquired two cardiac cycles per recording, one of which was selected by the observer for analysis. To calculate left ventricular longitudinal strain values, the endocardial borders were manually traced using the AVC automated speckle-tracking technique, and global longitudinal strain measurements were obtained from these standardized imaging planes [18].

Peak systolic longitudinal strain was measured offline from the apical four-, three- and two-chamber views using the Philips aCMQ software (version 9.0.2.443) after manual tracing of the endocardial border, with the systolic interval defined by automatically detected aortic valve closure. For each view, the six segmental endocardial strain values generated by the software were recorded, and the territorial mean strain of that view (A4C, A3C and A2C) was calculated as the arithmetic mean of its six segments. Global longitudinal strain was taken directly from the software (LV Global Endo Peak Longitudinal Strain) rather than recalculated by the investigators; this software-derived value corresponds to the mean of the three view-level values and agreed with it to within the rounding of the report. All strain values refer to the endocardial layer.

All strain analyses were performed by two experienced pediatric cardiologists who were blinded to group assignment and to all clinical and demographic information. The recordings of the patient and control groups were anonymized, randomly ordered, and analyzed in a single mixed sequence so that neither observer was aware of whether a given study belonged to a patient or to a control participant at the time of measurement. Because this study was based on retrospectively retrieved image loops, formal quantitative reproducibility testing (intra- and inter-observer) could not be completed during the study period; to minimize measurement variability, however, all analyses were performed on a single-vendor platform using a standardized tracing protocol under the blinded, mixed-sequence conditions described above.

### 2.4. Group Characteristics

Control participants were adolescents who presented to the pediatric cardiology outpatient clinic with minor complaints (chest pain or palpitations). Each underwent a complete cardiac evaluation—a detailed physical examination, standard 12-lead electrocardiography and transthoracic echocardiography, interpreted by a pediatric cardiologist—and only individuals with entirely normal echocardiographic end electrocardiographic findings and structurally normal hearts were included; no clinically relevant abnormalities were identified in this group.

Patients were excluded if the quality of the echocardiographic images was inadequate for myocardial strain analysis; if one or more of the apical four-, three-, or two-chamber views could not be obtained with sufficient quality; or if they had congenital or acquired heart disease, a history of cardiac surgery, or a known diagnosis of cardiomyopathy, heart failure, valvular disease, or arrhythmia. Patients with hypertension, chronic kidney disease, chronic pulmonary disease, hematologic disease, endocrine or metabolic disorders, genetic syndromes, neuromuscular diseases, or other chronic systemic conditions that could affect the cardiovascular system were also excluded. Patients with incomplete file data, including demographic information, substance-use history, electrocardiographic or echocardiographic records; unreliable measurements; or clinical conditions that could transiently affect cardiac function during examination, such as acute infection, fever, or hemodynamic instability, were likewise excluded. If more than one echocardiographic record was available for the same patient, the record with the most complete image quality and clinical information and the one closest to the study period was included in the analysis.

### 2.5. Ethics Committee Approval

This retrospective study was approved by the İzmir Buca Seyfi Demirsoy Training and Research Hospital Non-Interventional Clinical Research Ethics Committee under decision No.: 2025/410 (26 February 2025). This study was conducted in accordance with the ethical principles of the Declaration of Helsinki. Patient privacy and the protection of personal data were strictly observed throughout data processing; all records were anonymized, assigned identification numbers, and analyzed accordingly. The clinical and demographic data collected during this study were stored in an encrypted digital database to ensure the consistency of the statistical analyses.

### 2.6. Statistical Analysis

Normality was assessed within each group with the Shapiro–Wilk test. All continuous data are presented as median [Q1, Q3]. Normally distributed variables were compared with the independent-sample Student’s *t*-test, with the Welch correction when the Levene’s test indicated unequal variances, and the remainder with the Mann–Whitney U test. Effect sizes are reported as Hedges’ g and the rank-biserial correlation coefficient, respectively. Categorical variables were compared with the Pearson chi-square test or Fisher’s exact test.

Because the strain protocol generates many simultaneous comparisons, *p*-values were also adjusted by the Benjamini–Hochberg false discovery rate procedure within predefined families of variables (demographic, conventional echocardiographic, the 18 segmental strain values, and the territorial and global strain values). Both unadjusted and adjusted values are reported; a two-sided *p* < 0.05 was considered significant and is shown in bold.

Discriminative performance was assessed by receiver operating characteristic analysis, with the area under the curve reported with its Hanley–McNeil 95% confidence interval and a bootstrap interval from 2000 stratified resamples. Cut-offs were defined by the Youden index, and sensitivity, specificity, predictive values and accuracy were calculated at those thresholds. Because cut-off and performance were derived from the same sample, the analysis was internally validated by 1000 bootstrap resamples with out-of-bag testing, and optimism-corrected estimates are reported.

A multivariable binary logistic regression model was fitted to test whether the association between global longitudinal strain and group membership persisted after adjustment for age, sex, body mass index and heart rate. To characterize the dispersion of global longitudinal strain, the three territorial mean strains (A4C, A3C and A2C) were correlated with one another using the Pearson and Spearman correlation coefficients. A formal a priori sample size calculation was not performed, as this study included all eligible adolescents admitted during the recruitment period; the adequacy of the sample was therefore verified post hoc. Analyses were carried out in Python 3.12.3 (SciPy 1.17, scikit-learn 1.8, statsmodels 0.14).

## 3. Results

A total of 58 adolescents were analysed: 29 with substance-use disorder (patient group) and 29 age- and sex-matched healthy controls (control group). No a priori sample-size calculation was performed, as all eligible adolescents admitted during the recruitment period were included; the adequacy of the sample was therefore verified post hoc. With 29 participants per group, this study had 90% power at α = 0.05 (two-sided) to detect a standardised difference of 0.87—corresponding to approximately 1.6 percentage points of global longitudinal strain at the observed pooled standard deviation—and the difference actually observed (Hedges’ g = 2.00) substantially exceeded this threshold.

### 3.1. Demographic and Clinical Characteristics

Age did not differ between the groups (16 [16, 17] vs. 17 [16, 17] years, *p* = 0.198, p(FDR) = 0.277), nor did the sex distribution (17/29 vs. 19/29 male, *p* = 0.588), height (168.03 ± 7.59 vs. 170.93 ± 8.91 cm, *p* = 0.188), body mass index (20.5 [19.0, 22.1] vs. 22.6 [20.2, 24.8] kg/m^2^, *p* = 0.071), systolic blood pressure (113.0 [105.0, 121.0] vs. 116.0 [98.0, 129.0] mmHg, *p* = 0.809) or diastolic blood pressure (71.52 ± 7.75 vs. 70.28 ± 8.12 mmHg, *p* = 0.554). Body weight was higher in the patient group (59.0 [51.0, 65.0] vs. 64.0 [60.0, 73.0] kg, ***p*** **= 0.047**), although this difference did not persist after adjustment for multiple testing (p(FDR) = 0.165). Resting heart rate was lower in the patient group (80.48 ± 11.74 vs. 69.72 ± 12.52 beats/min, ***p*** **= 0.001**, **p(FDR) = 0.009**) (Table 1).

### 3.2. Substance-Use Characteristics of the Patient Group

Substance use had begun at a median age of 13 [12, 14] years, corresponding to a median exposure of 3 [2, 4] years. The median frequency of use was 4 [2, 7] days per week, and 13 adolescents (44.8%) reported daily use. Polysubstance use was recorded in 22 participants (75.9%). The most frequently used substances were pregabalin (12, 41.4%), methamphetamine (10, 34.5%), and synthetic cannabinoids (5, 17.2%), and the most common substance of initiation was cannabis (12, 41.4%). Cigarette smoking was reported by 22 patients (75.9%), and 7 (24.1%) were non-smokers; 16 (55.2%) smoked up to 20 cigarettes per day and 6 (20.7%) more than 20. Among smokers, the median consumption was 20 [20, 28] cigarettes per day (Table 2).

### 3.3. Conventional Echocardiographic and Doppler Findings

Transvalvular Doppler velocities did not differ between the groups (mitral 82.5 [71.8, 92.2] vs. 80.0 [71.3, 84.9] cm/s, *p* = 0.335; aortic 94.12 ± 17.08 vs. 94.41 ± 12.17 cm/s, *p* = 0.941; tricuspid 62.40 ± 9.92 vs. 65.39 ± 9.72 cm/s, *p* = 0.252; pulmonary 98.05 ± 17.89 vs. 94.84 ± 10.94 cm/s, *p* = 0.414), nor did end-diastolic wall thicknesses; LVPWd 0.79 ± 0.16 vs. 0.73 ± 0.13 cm, *p* = 0.183). End-systolic left ventricular dimensions and volumes were larger in the patient group (LVIDs 2.66 ± 0.36 vs. 2.91 ± 0.30 cm, ***p*** **= 0.005**, **p(FDR) = 0.026**; ESV 26.74 ± 8.91 vs. 33.17 ± 8.24 mL, ***p*** **= 0.006**, **p(FDR) = 0.026**), as was the end-diastolic right ventricular diameter (RVIDd 2.93 ± 0.44 vs. 3.17 ± 0.45 cm, ***p*** **= 0.043**, p(FDR) = 0.104), whereas the left ventricular end-diastolic measurements showed only non-significant trends (LVIDd 4.21 ± 0.48 vs. 4.46 ± 0.49 cm, *p* = 0.053; EDV 80.32 ± 20.98 vs. 91.79 ± 23.67 mL, *p* = 0.056). Left ventricular ejection fraction was lower in the patient group but remained within the normal range in every participant (66.92 ± 5.36 vs. 63.55 ± 4.91%, ***p*** **= 0.016**, p(FDR) = 0.050). Trace-to-mild mitral and tricuspid regurgitation were each present in 6 of 29 patients (20.7%) and in no control (both *p* = 0.023, Fisher’s exact test) (Table 3).

### 3.4. Segmental Longitudinal Strain

#### 3.4.1. Four-Chamber Results

In the apical four-chamber view, peak systolic longitudinal strain was less negative in the patient group in five of the six segments: mid anterolateral (−20.15 ± 4.83 vs. −16.31 ± 4.59%, ***p*** **= 0.003**, **p(FDR) = 0.007**); apical lateral (−25.23 ± 6.79 vs. −19.94 ± 6.30%, ***p*** **= 0.003**, **p(FDR) = 0.007**); apical septal (−24.89 ± 5.38 vs. −21.22 ± 5.49%, ***p*** **= 0.013**, **p(FDR) = 0.019**); mid inferoseptal (−21.83 ± 4.09 vs. −19.09 ± 3.18%, ***p*** **= 0.006**, **p(FDR) = 0.011**); basal inferoseptal (−17.28 ± 3.47 vs. −14.05 ± 4.68%, ***p*** **= 0.004**, **p(FDR) = 0.008**). No difference was demonstrated in the basal anterolateral segment (−14.9 [−17.4, −12.1] vs. −17.4 [−21.6, −13.4]%, *p* = 0.208). The territorial mean strain of this view was reduced in the patient group (−20.9 [−22.4, −19.3] vs. −18.0 [−19.3, −16.3]%, ***p*** **< 0.001**, **p(FDR) < 0.001**) (Table 4).

#### 3.4.2. Three-Chamber Results

In the apical three-chamber view, peak systolic longitudinal strain was less negative in the patient group in 5 of the six segments: basal anteroseptal (−16.7 [−22.4, −15.1] vs. −14.9 [−18.7, −12.6]%, ***p*** **= 0.035**, **p(FDR) = 0.045**); mid anteroseptal (−21.0 [−23.6, −19.1] vs. −17.8 [−21.4, −15.9]%, ***p*** **= 0.010**, **p(FDR) = 0.016**); apical anteroseptal (−24.89 ± 5.63 vs. −18.74 ± 5.01%, ***p*** **< 0.001**, **p(FDR) < 0.001**); apical inferolateral (−23.7 [−28.0, −18.6] vs. −18.3 [−21.2, −12.6]%, ***p*** **= 0.001**, **p(FDR) = 0.004**); mid inferolateral (−21.5 [−23.8, −19.7] vs. −15.7 [−18.3, −12.4]%, ***p*** **< 0.001**, **p(FDR) < 0.001**). No difference was demonstrated in the basal inferolateral segment (−18.2 [−22.6, −16.2] vs. −17.1 [−22.5, −14.2]%, *p* = 0.419). The territorial mean strain of this view was reduced in the patient group (−21.1 [−23.5, −19.5] vs. −16.8 [−19.3, −15.3]%, ***p*** **< 0.001**, **p(FDR) < 0.001**) (Table 5).

#### 3.4.3. Two-Chamber Results

In the apical two-chamber view, peak systolic longitudinal strain was less negative in the patient group in four of the six segments: basal anterior (−20.9 [−24.6, −15.6] vs. −15.0 [−19.8, −10.4]%, ***p*** **= 0.021**, **p(FDR) = 0.030**); apical anterior (−20.67 ± 6.62 vs. −14.16 ± 9.31%, ***p*** **= 0.003**, **p(FDR) = 0.007**); apical inferior (−26.34 ± 5.58 vs. −17.54 ± 6.26%, ***p*** **< 0.001**, **p(FDR) < 0.001**); mid inferior (−22.8 [−25.1, −19.2] vs. −14.9 [−20.3, −10.9]%, ***p*** **< 0.001**, **p(FDR) < 0.001**). No difference was demonstrated in the mid anterior or the basal inferior segments (−19.95 ± 6.06 vs. −17.28 ± 5.42%, *p* = 0.083 and −16.0 [−18.1, −12.1] vs. −16.0 [−19.1, −10.0]%, *p* = 0.901). The territorial mean strain of this view was reduced in the patient group (−20.51 ± 3.03 vs. −15.79 ± 3.46%, ***p*** **< 0.001**, **p(FDR) < 0.001**) (Table 6).

### 3.5. Territorial and Global Longitudinal Strain

**Figure 2:**
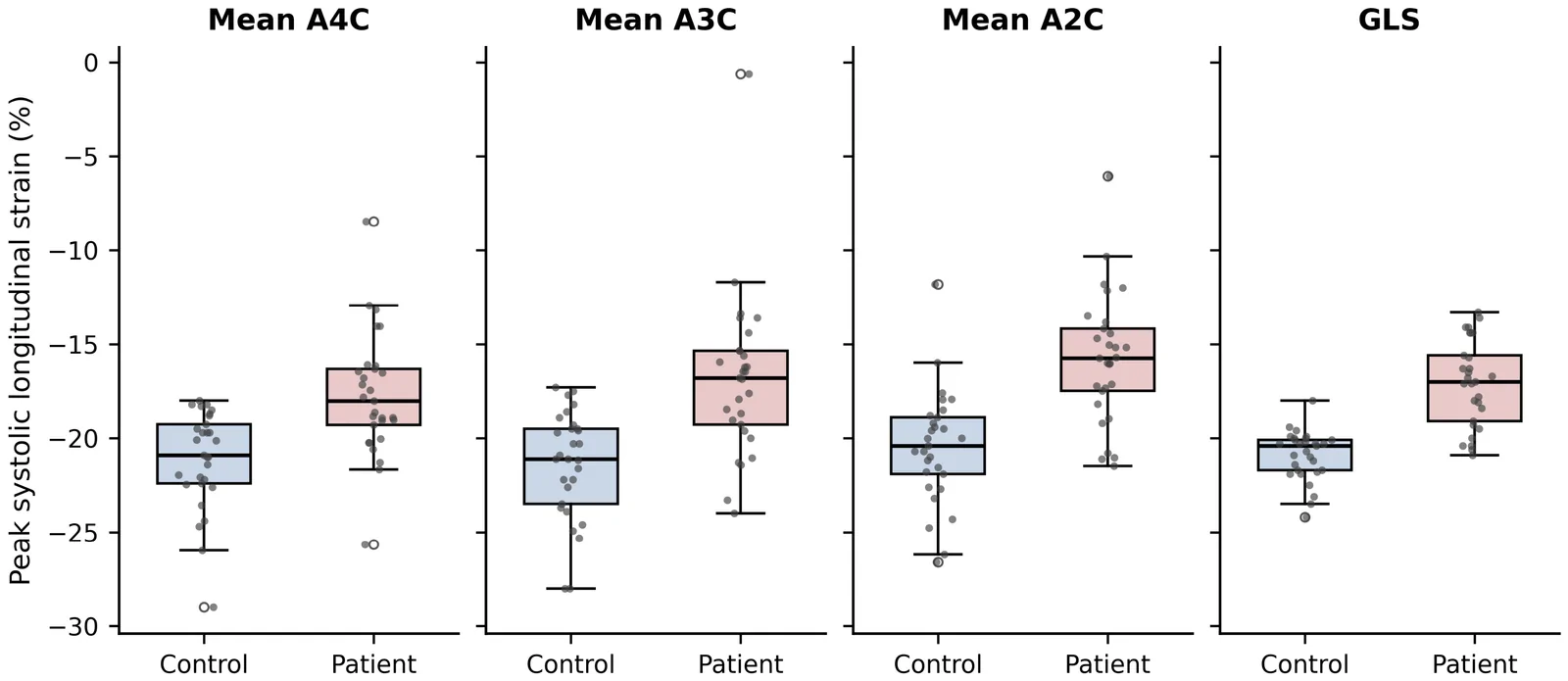
Distribution of territorial mean and global longitudinal strain in the control and patient groups. Boxes indicate the median and interquartile range; whiskers extend to 1.5 times the interquartile range; individual observations are superimposed.

Global longitudinal strain was less negative in the patient group (−20.91 ± 1.32 vs. −17.10 ± 2.30%, ***p*** **< 0.001**, **p(FDR) < 0.001**), an absolute difference of 3.8 percentage points (Hedges’ g = 2.00). The territorial means were reduced to a comparable degree: mean A4C (−20.9 [−22.4, −19.3] vs. −18.0 [−19.3, −16.3]%, ***p*** **< 0.001**, **p(FDR) < 0.001**); mean A3C (−21.1 [−23.5, −19.5] vs. −16.8 [−19.3, −15.3]%, ***p*** **< 0.001**, **p(FDR) < 0.001**); and mean A2C (−20.51 ± 3.03 vs. −15.79 ± 3.46%, ***p*** **< 0.001**, **p(FDR) < 0.001**). The reduction was of similar magnitude across all three apical views, indicating a diffuse rather than a regional impairment. The values of the patient group remained within the range conventionally regarded as normal for adolescents (Table 7, Figure 2).

**Figure 3:**
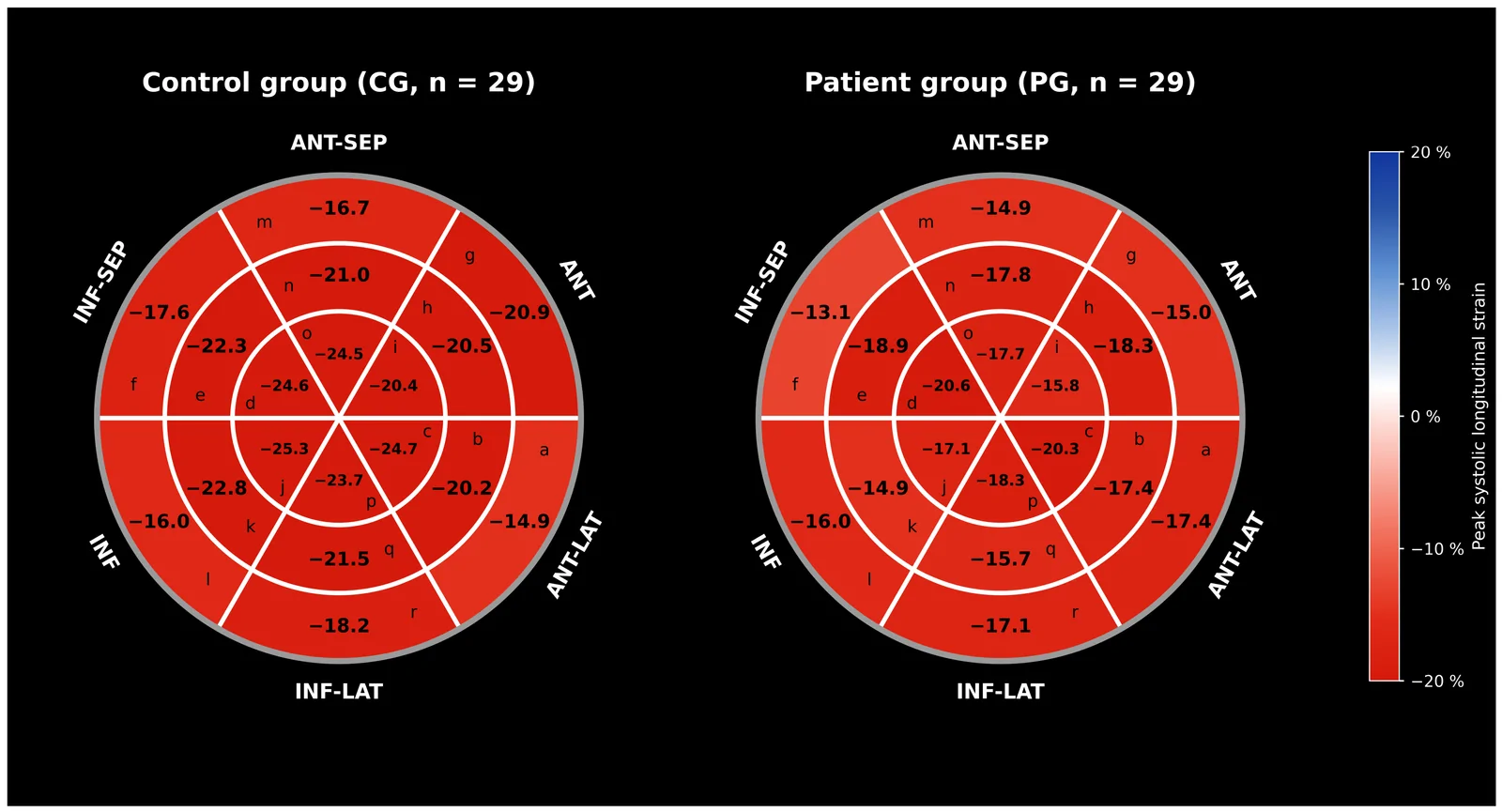
Eighteen-segment bullseye display of median peak systolic longitudinal strain in the control group (CG, **left**) and the patient group (PG, **right**), summarising the values presented in Table 4, Table 5 and Table 6. The outer, middle and inner rings correspond to the basal, mid-ventricular and apical levels, respectively; the six sectors correspond to the anteroseptal (ANT-SEP), anterior (ANT), anterolateral (ANT-LAT), inferolateral (INF-LAT), inferior (INF) and inferoseptal (INF-SEP) walls. Colour encodes the median strain value according to the scale bar, on which negative values (myocardial shortening) are displayed in red. The lower-case letter within each segment identifies the segment; segments are lettered by apical view, each series beginning at the anterior segment of that view: apical four-chamber view—a = basal anterolateral, b = mid anterolateral, c = apical lateral, d = apical septal, e = mid inferoseptal, f = basal inferoseptal; apical two-chamber view—g = basal anterior, h = mid anterior, i = apical anterior, j = apical inferior, k = mid inferior, l = basal inferior; apical three-chamber view—m = basal anteroseptal, n = mid anteroseptal, o = apical anteroseptal, p = apical inferolateral, q = mid inferolateral, r = basal inferolateral.

The bullseye maps (Figure 3) display the median value of each of the 18 segments in the two groups. The patient map is uniformly paler than the control map at all three ventricular levels and in all six walls, with the exception of the basal anterolateral and basal inferior segments, in which the median values of the two groups are identical or nearly so. The largest map differences are located in the apical and mid-ventricular segments of the inferior, inferolateral and anteroseptal walls.

### 3.6. Receiver Operating Characteristic Analysis

Global longitudinal strain discriminated patients from controls with an AUC of 0.913 (95% CI 0.836–0.991; bootstrap 95% CI 0.831–0.975; ***p*** **< 0.001**). At the Youden-optimal cut-off of −19.3%, the sensitivity was 79.3%, the specificity 96.6%, the positive predictive value 95.8%, the negative predictive value 82.4%, and the overall accuracy 87.9%. The territorial means yielded AUC values of 0.858 (95% CI 0.759–0.956) for mean A2C, 0.834 (95% CI 0.728–0.940) for mean A3C and 0.804 (95% CI 0.690–0.918) for mean A4C, at cut-offs of −17.5%, −16.8%, and −17.8% respectively; all four areas differed significantly from 0.5 (***p*** **< 0.001**) (Table 8).

The area under the curve is essentially free of optimism, which is expected when a single measured variable is used and no model is fitted; the small negative optimism values are sampling noise. The Youden-derived cut-offs behave differently: they are estimated and evaluated in the same data and are correspondingly optimistic. For global longitudinal strain the shrinkage is modest and the parameter remains serviceable, whereas the apparent specificity of 100% for the mean A3C and mean A4C thresholds falls by 16 and 21 percentage points, respectively, confirming that those values were an artefact of threshold selection. The cut-offs should accordingly be reported as exploratory, and external validation in an independent cohort should be identified as a requirement before any threshold is proposed for use (Table 9).

### 3.7. Multivariable Analysis

Age (odds ratio 0.37, 95% CI 0.12–1.18; *p* = 0.094), male sex (odds ratio 0.36, 95% CI 0.04–3.53; *p* = 0.383), and body mass index (odds ratio 0.83, 95% CI 0.54–1.26; *p* = 0.377) were not independently associated with group membership, whereas heart rate was (odds ratio 0.91 per beats/min, 95% CI 0.83–0.99; ***p*** **= 0.030**). Global longitudinal strain remained independently associated with membership of the patient group (odds ratio 6.98 per 1 percentage point less negative, 95% CI 1.72–28.36; ***p*** **= 0.007**). The model was significant overall (likelihood ratio ***p*** **< 0.001**; McFadden pseudo R^2^ = 0.644) (Table 10).

Inter-view correlations of the three territorial mean strain values differed between the groups (Table 11). In controls, the views showed no consistent positive correlation (mean Pearson’s r = −0.13), whereas in patients, they were positively correlated (mean r = +0.41). This difference explains the narrower dispersion of global longitudinal strain in the control group: because the mean of the three views inherits less variance when its components are uncorrelated, the standard deviation predicted from the component variances and their correlations (1.40%) closely matches the observed value (1.32%), and the corresponding figure in patients (predicted 2.84%, observed 2.30%) is likewise consistent (Table 12). The narrow control distribution therefore reflects the correlation structure of the averaged measurements rather than any additional source of variance reduction.

## 4. Discussion

According to the demographic, anthropometric, and hemodynamic data in our study, the patient and control groups were similar in terms of age, sex, body mass index, and basic cardiovascular characteristics, with no significant baseline demographic or hemodynamic differences between the groups. This is particularly important for the purpose of this study, as the similarity of the groups in terms of age, sex, body mass index, and basic hemodynamic characteristics enabled strain assessment to be performed more reliably and with less potential confounding in the relevant population. Therefore, the myocardial deformation differences observed between the groups are unlikely to have resulted solely from differences in these baseline characteristics and may more directly reflect the potential cardiac effects associated with substance use—effects that remained subclinical in the sense that ejection fraction and other conventional parameters, although differing modestly between groups, stayed within their normal reference ranges, with strain revealing the impairment most sensitively. Evaluation of the substance-use characteristics of the patient group revealed that substance use predominantly occurred in the form of polysubstance use; cannabis use was frequently accompanied by cigarette smoking and alcohol consumption, while pregabalin, methamphetamine, and synthetic cannabinoid use were also reported in some patients. Consistent with the substance-use pattern observed in our patient group, the literature has reported that polysubstance or mixed-substance use, rather than use limited to a single substance, is common among adolescents; cannabis, in particular, is frequently used together with other substances, especially cigarettes and alcohol [19]. This co-use may encompass concurrent use, in which substances are consumed on different occasions, and simultaneous use, in which they are taken during the same episode; among adolescents, the combined use of alcohol, tobacco, and cannabis may be more common than the use of these substances individually [20,21]. Accordingly, the co-occurrence of cannabis, cigarette, and alcohol use observed in our study is consistent with the general patterns of polysubstance use reported among adolescents. However, based on the available data, it cannot be determined with certainty in what order or how frequently these substances were used, or whether they were consumed during the same episode [3]. In our study, the co-occurrence of cigarette smoking and substance use could not be isolated in the patient group. Although this constitutes a confounding factor for this study, because the co-occurrence of smoking and substance use is extremely common in these patients in real life, we believe that our results remain applicable to clinical evaluation when both exposures are considered together. Among the substances reported in our study, pregabalin and methamphetamine were the most commonly used. Because substance-use patterns may vary regionally, the pattern observed in our patient group may reflect regional characteristics [22]. Nevertheless, given the predominance of polysubstance use, it would not be appropriate to attribute the observed cardiac effects to a single substance. The diverse substance-use patterns and widespread polysubstance use in the patient group make it difficult to attribute the cardiac effects to a specific substance and indicate the need for evaluation in larger samples according to substance type, duration of use, and dose.

When the heart rate findings in this study were evaluated, a significant difference was detected between the groups despite the heart rate being within the normal age-specific reference range. The heart rate was approximately 10 beats/min lower in the patient group. This difference may be related to the potential modulatory effects of chronic substance exposure on the autonomic nervous system or to compensatory adaptations to changes in cardiovascular hemodynamics; however, the available cross-sectional data do not allow these mechanisms to be confirmed [23,24]. Substance use may alter heart rate by increasing sympathetic activity; particularly with synthetic cannabinoids, bradycardia may also occur, in addition to tachycardia and hypertension, depending on the type and dose of the substance used [13,25]. Therefore, heart rate should not be considered a direct cause of strain impairment, but rather a possible indicator of autonomic and cardiovascular changes that may accompany substance use. In particular, the predominance of polysubstance use and the limited information regarding the timing and dosage of substance use make it difficult to attribute the observed bradycardia to a specific substance or directly to impaired myocardial deformation [13,25]. Accordingly, the difference in heart rate should be considered a potential confounder that must be taken into account when interpreting the strain findings; however, based on the available cross-sectional data, it should not be claimed that the reduction in heart rate caused myocardial strain impairment.

The conventional echocardiographic and Doppler findings in our study were also similar between the patient and control groups and generally remained within normal reference ranges [26]. Although statistically significant differences were detected in LVIDs values, the fact that these values remained within age-specific normal reference ranges suggests that these differences should not be interpreted as clinically abnormal findings in isolation [27]. However, strain echocardiography may contribute to the detection of subclinical myocardial involvement that is not apparent on conventional echocardiography [28]. This technique, which has increasingly become an indispensable tool for assessing myocardial deformation in the literature, enables the sensitive and multidimensional analysis of cardiac function; therefore, it supports efforts to incorporate deformation imaging into clinical guidelines [29]. In this context, the primary aim of our study was to use strain echocardiography to evaluate, at an early stage, myocardial abnormalities that may not be detected by conventional parameters in young patients with substance dependence and to investigate the potential of the data obtained using this method to serve as a prognostic marker in long-term cardiac follow-up [30,31].

This early myocardial deformation loss, detectable by strain echocardiography while ejection fraction is preserved, may represent a potential long-term risk marker in young individuals with substance dependence. Data specifically addressing adults with a longer duration of substance exposure than our adolescent cohort are limited, but studies in adult substance users are consistent with our findings: reduced longitudinal strain despite preserved ejection fraction has been reported in asymptomatic cocaine users and in opioid users, and abnormal global longitudinal strain has been described in young smokers [32,33,34]. That comparable deformation abnormalities are already detectable in our adolescents after a median exposure of only three years suggests these early changes may begin early and could progress with longer exposure, although prospective longitudinal data linking adolescent findings to adult outcomes are not yet available [10,35].

The most important findings of our study were the results obtained from the global and segmental longitudinal strain analyses. In these analyses, particularly in the global longitudinal strain values, a more pronounced decline than expected was observed. However, the fact that strain values vary with age in pediatric patients presents an important challenge in interpreting these findings [36]. Therefore, age-specific reference ranges should be taken into account when evaluating strain results. In this regard, studies in the literature have shown that myocardial longitudinal strain values exhibit a weak but significant nonlinear relationship with age; particularly after the age of 5 years, strain values progressively become less negative toward adolescence. Accordingly, the use of separate reference values for each age group has been recommended [36]. Because GLS in children is age- and platform-dependent, adolescents may exhibit different reference values across studies [37]. In the 14–18-year age range, Romanowicz et al. reported a GLS of −21 ± 3%, whereas Cantinotti et al. reported a value of −24.0 ± 2.3% for the 11–18-year age range [36]. In our study, the mean GLS was −20.4%, indicating a less negative value than those reported in both reference populations. This difference should nevertheless be interpreted cautiously because the studies used different age ranges, software platforms, and analysis protocols. The QLAB and AutoStrain values reported in the Romanowicz series for the 14–18-year age group similarly demonstrate that strain values vary according to the software platform and may become progressively less negative during adolescence, despite differences of several percentage points between software platforms [36]. Similarly, in the series by Kamel et al. included in the same nomogram, LV GLS in children older than 5 years was reported to decline to −19.9 ± 0.9 and to become progressively less negative with age [36]. In our study, territorial mean strain values obtained from apical four-, three-, and two-chamber views, together with GLS reported by the software, were measured using the aCMQ software integrated into the Philips Affiniti 50 G device in 29 healthy controls who were comparable to the patient group in terms of age and sex. These values showed a distribution consistent with the reference distribution defined by Romanowicz for the 14–18-year age range and with the software-specific norms recommended for adolescents. This finding confirms that our control group had adequate representational capacity as a healthy reference population appropriate for the age range and myocardial segments. Accordingly, because the patient group was assessed using the same protocol and software, the reduction in strain—characterized by an average decrease of 3.4 percentage points in global longitudinal strain and significant reductions in numerous segments—appears more likely to reflect true early myocardial pathology associated with substance use than natural variation related to age, body size, or the measurement method. Nevertheless, this interpretation should be considered within the limitations of the cross-sectional design and the small sample size.

The significant reduction in most segmental global longitudinal strain measurements suggests diffuse left ventricular involvement rather than injury confined to a single coronary territory. This pattern may be consistent with the toxic, autonomic, or metabolic effects of substance use, although the cross-sectional design cannot establish a specific mechanism or demonstrate direct myocyte toxicity [28,33,34]. Reporting territorial strain values alongside GLS demonstrates that the impairment was distributed across the three apical views and was not driven by a single region. Longitudinal studies should determine whether these abnormalities change after continued or discontinued substance use and whether they progress to structural cardiomyopathy. Because circumferential deformation may compensate for reduced longitudinal strain while ejection fraction remains preserved, longitudinal, circumferential, and radial strain should be evaluated together rather than relying on GLS alone [11].

In our study, the global longitudinal strain presented in Table 8 demonstrated the highest discriminative power among the four deformation parameters, and the ranking of the regional mean values was consistent with the segmental analysis; within this ranking, the apical two-chamber view provided the strongest contribution. This finding should be interpreted cautiously, taking into account the characteristics and limited size of the study sample. Compared with segmental strain measurements, the discriminative specificity of global strain appeared to be higher; however, this observation needs to be validated in larger samples. The threshold values obtained in our study should not be regarded as pathological cutoff values because of the limited sample size; rather, they should be considered exploratory and hypothesis-generating findings specific to this study population. A formal comparison of the correlated areas under the curves was not performed because of the strong collinearity among the parameters and the insufficient sample size to obtain informative results from such an analysis. The apparent 100% specificity observed at the mean A3C and mean A4C threshold values was likely an artifact of threshold selection in the small sample and was not reproduced during internal validation. Studies involving larger samples may help determine which imaging window is more effective in segmental strain echocardiography. Nevertheless, it should be considered that global strain may be affected in young individuals with substance dependence and that this impairment may reflect a pattern of diffuse myocardial involvement. In this context, myocardial deformation parameters, particularly in cases in which ejection fraction remains within the normal range, may be important in clinical decision-making as more sensitive indicators of subclinical myocardial injury [18,33].

The persistence of the association between strain and group after multivariable adjustment is an important finding of the model, because longitudinal strain may be affected by both loading conditions and heart rate, and the groups differed in resting heart rate [38]. Accordingly, this finding suggests that the difference in deformation is unlikely to be merely a hemodynamic by-product of a slower heart rate. The independent contribution of heart rate to the model should not be overinterpreted, as it may reflect the autonomic effects of substance use, withdrawal, or the inpatient setting in which the patients were evaluated. The model is exploratory in nature: with 29 cases and 5 covariates, it falls below the recommended evaluation threshold [39]. The wide confidence interval of the odds ratio estimated for strain also reflects the limited sample size. Therefore, the odds ratio should be interpreted not as evidence of an “independent association,” but as a sample-specific estimate of association; its magnitude should not be regarded as a calibrated estimate.

Strain echocardiographic measurements, which reflect myocardial contractility more sensitively than other systolic parameters, may play a critical complementary role in detecting early myocardial dysfunction in substance users before structural damage or a marked decline in ejection fraction develops [40]. In contrast to conventional echocardiographic methods, this advanced imaging approach enables a more sensitive characterization of reductions in myocardial functional reserve and changes in regional contractility before structural abnormalities become clinically apparent [18,32]. Indeed, the detection of reduced strain and strain rate values compared with healthy controls even in asymptomatic cocaine users with preserved ejection fraction suggests that these parameters may reveal subclinical myocardial involvement [34]. Nevertheless, because strain echocardiographic measurements may be influenced by preload, afterload, and heart rate, these measurements should not be interpreted in isolation as definitive indicators of myocardial contractility but rather in conjunction with conventional echocardiographic parameters, clinical findings, and, whenever possible, serial assessments. Therefore, the use of strain analyses as part of a comprehensive echocardiographic evaluation may represent a promising approach for identifying early cardiac involvement in substance users and individuals at risk of developing structural cardiomyopathy, although this approach requires confirmation in future studies.

## 5. Conclusions

This study suggests that evaluating cardiac function in young individuals with substance and tobacco dependence using strain imaging techniques, in addition to routine echocardiographic parameters, may represent a promising approach for the early detection of early myocardial injury. These advanced imaging methods enable an objective and quantitative assessment of myocardial deformation, particularly during the early stages when conventional indicators of systolic function remain within normal reference ranges, and may therefore provide valuable information to support early cardioprotective interventions. In addition, monitoring the progression of substance-use-related myocardial injury over time in adolescents may be important for reducing long-term cardiac morbidity and objectively assessing treatment response. Future large-scale, prospective studies should clarify the clinical value of these non-invasive imaging techniques for prognostic assessment and follow-up; the evidence generated may contribute to the development of a standardised monitoring protocol for young individuals with substance dependence.

## 6. Limitations

This study has several limitations. The sample was small and drawn from a single centre, limiting precision and generalisability, particularly to female adolescents and to community populations with less severe use. The cross-sectional design precludes causal inference and cannot establish whether the impairment precedes, accompanies, or follows substance use, or whether it is reversible with abstinence. Cigarette smoking was nearly universal among patients and absent among controls; so, tobacco could not be separated statistically from the substances studied; the findings should therefore be attributed to the composite exposure rather than to any single agent. The cohort was also pharmacologically heterogeneous, with most participants reporting polysubstance use, precluding analysis by substance class.

Exposure was characterised by self-report, in coarse units and without biochemical verification; the absence of a dose–response relationship may therefore reflect a threshold effect, measurement error, or limited power rather than a true absence. Several covariates were unavailable—psychiatric comorbidity, psychotropic medication, alcohol, physical activity, diastolic function, right ventricular deformation, and biomarkers of myocardial injury; so, residual confounding cannot be excluded and the underlying mechanism remains uncertain. Longitudinal strain is also load- and heart-rate-dependent; although resting heart rate was included in the multivariable model, loading conditions were not otherwise characterised, and because imaging was performed during early abstinence, the findings cannot be extrapolated to acute intoxication or sustained recovery.

Finally, all measurements were obtained on a single vendor platform; so, the absolute values and thresholds are vendor-specific and not interchangeable across systems. Formal reproducibility (ICC, CV, Bland–Altman) was not tested, and a small proportion of segments were not physiologically interpretable; the 3.4-percentage-point difference could thus approach reported interobserver variability, although its directional concordance across all three views and most segments and its persistence after FDR and multivariable adjustment argue against random error. Because thresholds were derived and evaluated in the same matched case–control sample, they appeared optimistic on internal validation and reflect group separability rather than screening performance; external, multi-platform validation is therefore required before clinical use.

## Figures and Tables

**Table 1:** Demographic, anthropometric and haemodynamic characteristics of the study groups.

Variable	Control Group (n = 29)	Patient Group (n = 29)	Effect Size (95% CI)	*p*	p (FDR)
Age, years *(median [Q1, Q3])*	16 [16, 17]	17 [16, 17]	0.18 (−0.09 to 0.44)	0.198	0.277
Male sex *(n (%))*	17 (58.6)	19 (65.5)	—	0.588	—
Body weight, kg *(median [Q1, Q3])*	59.0 [51.0, 65.0]	64.0 [60.0, 73.0]	0.30 (−0.00 to 0.57)	**0.047**	0.165
Height, cm *(mean ± SD)*	168.03 ± 7.59	170.93 ± 8.91	0.35 (−0.17 to 0.86)	0.188	0.277
Body mass index, kg/m^2^ *(median [Q1, Q3])*	20.5 [19.0, 22.1]	22.6 [20.2, 24.8]	0.28 (−0.03 to 0.56)	0.071	0.166
Systolic blood pressure, mmHg *(median [Q1, Q3])*	113.0 [105.0, 121.0]	116.0 [98.0, 129.0]	0.04 (−0.29 to 0.34)	0.809	0.809
Diastolic blood pressure, mmHg *(mean ± SD)*	71.52 ± 7.75	70.28 ± 8.12	−0.15 (−0.66 to 0.35)	0.554	0.646
Heart rate, beats/min *(mean ± SD)*	80.48 ± 11.74	69.72 ± 12.52	−0.87 (−1.41 to −0.34)	**0.001**	**0.009**

Data are shown in the format matching the between-group test applied to each variable and indicated in parentheses after the variable name: variables compatible with a normal distribution in both groups are given as mean ± standard deviation (mean ± SD) and were compared with the *t* test, and the remaining variables as median [first quartile, third quartile] (median [Q1, Q3]) and were compared with the Mann–Whitney U test. Categorical data are n (%) and were compared with the Pearson chi-square test. Effect sizes are Hedges g for variables compared with the *t* test and the rank-biserial correlation for those compared with the Mann–Whitney U test, expressed for the patient group relative to the control group, each with its 95% confidence interval. p (FDR) = Benjamini–Hochberg adjusted *p* value within the demographic family. Significant values (*p* < 0.05) are shown in bold. Colored an italicized text indicates explanations of the values.

**Table 2:** Substance use and smoking profile of the patient group. Continuous data are median [Q1, Q3]; categorical data are n (%). These variables were recorded only in the patient group (all control participants were substance-naive and non-smokers by definition) and were therefore not subjected to between-group testing.

Variable	Patient Group (n = 29)
Age at first substance use, years	13 [12, 14]
Duration of substance exposure, years	3 [2, 4]
Frequency of use, days per week	4 [2, 7]
Daily use (7 days per week), n (%)	13 (44.8)
Interval since last use, days	7 [2, 21]
Polysubstance use (≥2 substances), n (%)	22 (75.9)
Current cigarette smoking, n (%)	22 (75.9)
Non-smokers, n (%)	7 (24.1)
≤20 cigarettes per day, n (%)	16 (55.2)
>20 cigarettes per day, n (%)	6 (20.7)
Cigarettes per day, smokers only	20 [20, 28]
Most frequently used substance—Pregabalin, n (%)	12 (41.4)
Most frequently used substance—Methamphetamine, n (%)	10 (34.5)
Most frequently used substance—Synthetic cannabinoid, n (%)	5 (17.2)
Most frequently used substance—Cannabis, n (%)	1 (3.4)
Most frequently used substance—Ecstasy, n (%)	1 (3.4)
First substance ever used—Cannabis, n (%)	12 (41.4)
First substance ever used—Pregabalin, n (%)	11 (37.9)
First substance ever used—Methamphetamine, n (%)	5 (17.2)
First substance ever used—Synthetic cannabinoid, n (%)	1 (3.4)

**Table 3:** Conventional two-dimensional, M-mode and Doppler echocardiographic parameters.

Variable	Control Group (n = 29)	Patient Group (n = 29)	Effect Size (95% CI)	*p*	p (FDR)
Mitral valve velocity, cm/s *(median [Q1, Q3])*	82.5 [71.8, 92.2]	80.0 [71.3, 84.9]	−0.15 (−0.44 to 0.16)	0.335	0.435
Aortic valve velocity, cm/s *(mean ± SD)*	94.12 ± 17.08	94.41 ± 12.17	0.02 (−0.49 to 0.53)	0.941	0.941
Tricuspid valve velocity, cm/s *(mean ± SD)*	62.40 ± 9.92	65.39 ± 9.72	0.30 (−0.21 to 0.81)	0.252	0.364
Pulmonary valve velocity, cm/s *(mean ± SD)*	98.05 ± 17.89	94.84 ± 10.94	−0.21 (−0.72 to 0.30)	0.414	0.490
LVIDd, cm *(mean ± SD)*	4.21 ± 0.48	4.46 ± 0.49	0.51 (−0.00 to 1.03)	0.053	0.104
LVIDs, cm *(mean ± SD)*	2.66 ± 0.36	2.91 ± 0.30	0.77 (0.24 to 1.29)	**0.005**	**0.026**
LVPWd, cm *(mean ± SD)*	0.79 ± 0.16	0.73 ± 0.13	−1.18 (−1.73 to −0.63)	0.183	0.297
RVIDd, cm *(mean ± SD)*	2.93 ± 0.44	3.17 ± 0.45	0.54 (0.02 to 1.05)	**0.043**	0.104
LV ejection fraction, % *(mean ± SD)*	66.92 ± 5.36	63.55 ± 4.91	0.51 (−0.01 to 1.02)	**0.016**	0.050
LV end-diastolic volume, mL *(mean ± SD)*	80.32 ± 20.98	91.79 ± 23.67	0.74 (0.21 to 1.26)	0.056	0.104
LV end-systolic volume, mL *(mean ± SD)*	26.74 ± 8.91	33.17 ± 8.24	−0.65 (−1.17 to −0.13)	**0.006**	**0.026**

Data are shown in the format matching the between-group test applied to each variable and indicated in parentheses after the variable name: variables compatible with a normal distribution in both groups are given as mean ± standard deviation (mean ± SD) and were compared with the *t* test, and the remaining variables as median [first quartile, third quartile] (median [Q1, Q3]) and were compared with the Mann–Whitney U test. Effect sizes are Hedges g for variables compared with the *t* test and the rank-biserial correlation for those compared with the Mann–Whitney U test, expressed for the patient group relative to the control group, each with its 95% confidence interval. p (FDR) = Benjamini–Hochberg adjusted *p* value within the conventional echocardiography family (13 comparisons). Significant values (*p* < 0.05) are shown in bold. Colored an italicized text indicates explanations of the values. LVPWd = left ventricular posterior wall thickness at end-diastole; LVIDd/LVIDs = left ventricular internal diameter at end-diastole/end-systole; RVIDd = right ventricular internal diameter at end-diastole; LV: Left ventricular.

**Table 4:** Segmental peak systolic longitudinal strain in the apical four-chamber view (%).

Variable	Control Group (n = 29)	Patient Group (n = 29)	Effect Size	*p*	p (FDR)
Basal anterolateral *(median [Q1, Q3])*	−14.9 [−17.4, −12.1]	−17.4 [−21.6, −13.4]	−0.19	0.208	0.234
Mid anterolateral *(mean ± SD)*	−20.15 ± 4.83	−16.31 ± 4.59	0.80	**0.003**	**0.007**
Apical lateral *(mean ± SD)*	−25.23 ± 6.79	−19.94 ± 6.30	0.80	**0.003**	**0.007**
Apical septal *(mean ± SD)*	−24.89 ± 5.38	−21.22 ± 5.49	0.67	**0.013**	**0.019**
Mid inferoseptal *(mean ± SD)*	−21.83 ± 4.09	−19.09 ± 3.18	0.74	**0.006**	**0.011**
Basal inferoseptal *(mean ± SD)*	−17.28 ± 3.47	−14.05 ± 4.68	0.77	**0.004**	**0.008**
Mean A4C longitudinal strain *(median [Q1, Q3])*	−20.9 [−22.4, −19.3]	−18.0 [−19.3, −16.3]	0.61	**<0.001**	**<0.001**

Data are shown in the format matching the between-group test applied to each variable and indicated in parentheses after the variable name: variables compatible with a normal distribution in both groups are given as mean ± standard deviation (mean ± SD) and were compared with the *t* test, and the remaining variables as median [first quartile, third quartile] (median [Q1, Q3]) and were compared with the Mann–Whitney U test. All values are peak systolic longitudinal strain expressed in per cent; more negative values denote greater myocardial shortening. Effect sizes are Hedges g for variables compared with the *t* test and the rank-biserial correlation for those compared with the Mann–Whitney U test, expressed for the patient group relative to the control group. p (FDR) for the individual segments refers to Benjamini–Hochberg adjustment across all 18 segments; for the territorial mean the adjustment was performed within the corresponding view. Significant values (*p* < 0.05) are shown in bold. Colored an italicized text indicates explanations of the values.

**Table 5:** Segmental peak systolic longitudinal strain in the apical three-chamber (long-axis) view (%).

Variable	Control Group (n = 29)	Patient Group (n = 29)	Effect Size (95% CI)	*p*	p (FDR)
Basal anteroseptal *(median [Q1, Q3])*	−16.7 [−22.4, −15.1]	−14.9 [−18.7, −12.6]	0.32 (0.01 to 0.59)	**0.035**	**0.045**
Mid anteroseptal *(median [Q1, Q3])*	−21.0 [−23.6, −19.1]	−17.8 [−21.4, −15.9]	0.40 (0.10 to 0.65)	**0.010**	**0.016**
Apical anteroseptal *(mean ± SD)*	−24.89 ± 5.63	−18.74 ± 5.01	1.14 (0.59 to 1.69)	**<0.001**	**<0.001**
Apical inferolateral *(median [Q1, Q3])*	−23.7 [−28.0, −18.6]	−18.3 [−21.2, −12.6]	0.50 (0.20 to 0.72)	**0.001**	**0.004**
Mid inferolateral *(median [Q1, Q3])*	−21.5 [−23.8, −19.7]	−15.7 [−18.3, −12.4]	0.64 (0.37 to 0.83)	**<0.001**	**<0.001**
Basal inferolateral *(median [Q1, Q3])*	−18.2 [−22.6, −16.2]	−17.1 [−22.5, −14.2]	0.12 (−0.18 to 0.43)	0.419	0.443
Mean A3C longitudinal strain *(median [Q1, Q3])*	−21.1 [−23.5, −19.5]	−16.8 [−19.3, −15.3]	0.67 (0.41 to 0.84)	**<0.001**	**<0.001**

Data are shown in the format matching the between-group test applied to each variable and indicated in parentheses after the variable name: variables compatible with a normal distribution in both groups are given as mean ± standard deviation (mean ± SD) and were compared with the *t* test, and the remaining variables as median [first quartile, third quartile] (median [Q1, Q3]) and were compared with the Mann–Whitney U test. All values are peak systolic longitudinal strain expressed in per cent; more negative values denote greater myocardial shortening. Effect sizes are Hedges g for variables compared with the *t* test and the rank-biserial correlation for those compared with the Mann–Whitney U test, expressed for the patient group relative to the control group. p (FDR) for the individual segments refers to Benjamini–Hochberg adjustment across all 18 segments; for the territorial mean the adjustment was performed within the corresponding view. Significant values (*p* < 0.05) are shown in bold. Colored an italicized text indicates explanations of the values.

**Table 6:** Segmental peak systolic longitudinal strain in the apical two-chamber view (%).

Variable	Control Group (n = 29)	Patient Group (n = 29)	Effect Size (95% CI)	*p*	p (FDR)
Basal anterior *(median [Q1, Q3])*	−20.9 [−24.6, −15.6]	−15.0 [−19.8, −10.4]	0.35 (0.05 to 0.62)	**0.021**	**0.030**
Mid anterior *(mean ± SD)*	−19.95 ± 6.06	−17.28 ± 5.42	0.46 (−0.06 to 0.97)	0.083	0.099
Apical anterior *(mean ± SD)*	−20.67 ± 6.62	−14.16 ± 9.31	0.80 (0.27 to 1.32)	**0.003**	**0.007**
Apical inferior *(mean ± SD)*	−26.34 ± 5.58	−17.54 ± 6.26	1.46 (0.89 to 2.04)	**<0.001**	**<0.001**
Mid inferior *(median [Q1, Q3])*	−22.8 [−25.1, −19.2]	−14.9 [−20.3, −10.9]	0.59 (0.34 to 0.79)	**<0.001**	**<0.001**
Basal inferior *(median [Q1, Q3])*	−16.0 [−18.1, −12.1]	−16.0 [−19.1, −10.0]	0.02 (−0.29 to 0.33)	0.901	0.901
Mean A2C longitudinal strain *(mean ± SD)*	−20.51 ± 3.03	−15.79 ± 3.46	1.43 (0.86 to 2.01)	**<0.001**	**<0.001**

Data are shown in the format matching the between-group test applied to each variable and indicated in parentheses after the variable name: variables compatible with a normal distribution in both groups are given as mean ± standard deviation (mean ± SD) and were compared with the *t* test, and the remaining variables as median [first quartile, third quartile] (median [Q1, Q3]) and were compared with the Mann–Whitney U test. All values are peak systolic longitudinal strain expressed in per cent; more negative values denote greater myocardial shortening. Effect sizes are Hedges g for variables compared with the *t* test and the rank-biserial correlation for those compared with the Mann–Whitney U test, expressed for the patient group relative to the control group, each with its 95% confidence interval. p (FDR) for the individual segments refers to Benjamini–Hochberg adjustment across all 18 segments; for the territorial mean the adjustment was performed within the corresponding view. Significant values (*p* < 0.05) are shown in bold. Colored an italicized text indicates explanations of the values.

**Table 7:** Territorial and global longitudinal strain (%).

Variable	Control Group (n = 29)	Patient Group (n = 29)	Effect Size (95% CI)	*p*	p (FDR)
Mean A4C longitudinal strain *(median [Q1, Q3])*	−20.9 [−22.4, −19.3]	−18.0 [−19.3, −16.3]	0.61 (0.35 to 0.80)	**<0.001**	**<0.001**
Mean A3C longitudinal strain *(median [Q1, Q3])*	−21.1 [−23.5, −19.5]	−16.8 [−19.3, −15.3]	0.67 (0.42 to 0.84)	**<0.001**	**<0.001**
Mean A2C longitudinal strain *(mean ± SD)*	−20.51 ± 3.03	−15.79 ± 3.46	1.43 (0.86 to 2.01)	**<0.001**	**<0.001**
Global longitudinal strain *(mean ± SD)*	−20.91 ± 1.32	−17.10 ± 2.30	2.00 (1.37 to 2.63)	**<0.001**	**<0.001**

Data are shown in the format matching the between-group test applied to each variable and indicated in parentheses after the variable name: variables compatible with a normal distribution in both groups are given as mean ± standard deviation (mean ± SD) and were compared with the *t* test, and the remaining variables as median [first quartile, third quartile] (median [Q1, Q3]) and were compared with the Mann–Whitney U test. Effect sizes are Hedges g for variables compared with the *t* test and the rank-biserial correlation for those compared with the Mann–Whitney U test, expressed for the patient group relative to the control group, each with its 95% confidence interval. p (FDR) = Benjamini–Hochberg adjusted *p* value within the territorial and global strain family. Significant values (*p* < 0.05) are shown in bold. Colored an italicized text indicates explanations of the values. A4C/A3C/A2C = apical four-, three- and two-chamber view. Global longitudinal strain is the value reported by the analysis software.

**Table 8:** Diagnostic performance of strain and conventional echocardiographic parameters in discriminating adolescents with substance-use disorder from healthy controls. AUC = area under the receiver operating characteristic curve; CI = confidence interval (Hanley–McNeil); PPV = positive predictive value; NPV = negative predictive value. The cut-off is the value maximising the Youden index; ≥ indicates that values equal to or above the threshold (i.e., less negative strain) identify the patient group, ≤ that values at or below the threshold do so. p refers to the null hypothesis that AUC = 0.5; significant values in bold. Sensitivity, specificity and accuracy are apparent (in-sample) estimates; the internally validated counterparts are given in Table 9. Predictive values apply to the 1:1 design of this sample.

Parameter	AUC (95% CI)	*p*	Cut-Off	Sensitivity, %	Specificity, %	PPV, %	NPV, %	Accuracy, %
Global longitudinal strain	0.913 (0.836–0.991)	**<0.001**	≥−19.3	79.3	96.6	95.8	82.4	87.9
Mean A2C longitudinal strain	0.858 (0.759–0.956)	**<0.001**	≥−17.5	75.9	93.1	91.7	79.4	84.5
Mean A3C longitudinal strain	0.834 (0.728–0.940)	**<0.001**	≥−16.8	55.2	100.0	100.0	69.0	77.6
Mean A4C longitudinal strain	0.804 (0.690–0.918)	**<0.001**	≥−17.8	48.3	100.0	100.0	65.9	74.1

**Table 9:** Internal validation of the receiver operating characteristic analysis by 1000 bootstrap resamples with out-of-bag testing. In each bootstrap resample, the Youden cut-off was re-derived and applied to the observations not drawn into that resample; the tabulated validated values are the means of the resulting out-of-bag estimates. The optimism-corrected AUC is the apparent AUC minus the mean difference between the resample and the out-of-bag AUC.

Parameter	Apparent AUC	Optimism-Corrected AUC	Sensitivity, % (Apparent → Validated)	Specificity, % (Apparent → Validated)	Validated Accuracy, %
Global longitudinal strain	0.913	0.920	79.3 → 76.9	96.6 → 93.4	85.1
Mean A2C longitudinal strain	0.858	0.855	75.9 → 71.6	93.1 → 88.4	79.9
Mean A3C longitudinal strain	0.834	0.837	55.2 → 59.7	100.0 → 84.3	72.0
Mean A4C longitudinal strain	0.804	0.806	48.3 → 57.4	100.0 → 79.3	68.4

**Table 10:** Multivariable binary logistic regression model for membership of the patient group. Model chi-square *p* < 0.001; McFadden pseudo R^2^ = 0.644. CI = confidence interval; significant values in bold. A positive coefficient for global longitudinal strain denotes that less negative (i.e., more impaired) strain increases the odds of belonging to the patient group.

Variable	Odds Ratio	95% CI	*p*
Age (per year)	0.37	0.12–1.18	0.094
Male sex	0.36	0.04–3.53	0.383
Body mass index (per kg/m^2^)	0.83	0.54–1.26	0.377
Heart rate (beats/min)	0.91	0.83–0.99	**0.030**
Global longitudinal strain (per 1% less negative)	6.98	1.72–28.36	**0.007**

**Table 11:** Inter-view correlations of territorial longitudinal strain and the resulting dispersion of global longitudinal strain. Pearson’s and Spearman’s correlation coefficients between the three apical territorial mean strain values, computed separately within each group. A4C, A3C and A2C denote the apical four-, three- and two-chamber views.

View Pair	Pearson’s r	*p*	Spearman’s ρ	*p*
**Control group (n = 29)**				
A4C–A3C	+0.155	0.422	+0.090	0.641
A4C–A2C	−0.103	0.596	−0.043	0.825
A3C–A2C	**−0.429**	**0.020**	−0.276	0.147
Mean of the three coefficients	−0.125			
**Patient group (n = 29)**				
A4C–A3C	+0.252	0.187	**+0.443**	**0.016**
A4C–A2C	**+0.690**	**<0.001**	**+0.550**	**0.002**
A3C–A2C	+0.292	0.124	**+0.431**	**0.020**
Mean of the three coefficients	+0.411			

Values are correlation coefficients with two-sided *p* values; coefficients significant at *p* < 0.05 are shown in bold. In the control group, the three views are essentially uncorrelated (mean r = −0.125), whereas in the patient group, they are positively correlated (mean r = +0.411).

**Table 12:** Standard deviation of global longitudinal strain: observed values versus predictions from inter-view correlations.

Standard Deviation of Global Longitudinal Strain (%)	Control	Patient
Predicted from the three views and their correlations	1.40	2.84
Observed (software-derived global value)	1.32	2.30
Mean of all 18 segments	1.37	2.84
Expected if the three views were independent	1.64	2.14

The predicted standard deviation is obtained from the variance in a mean of correlated variables, Var(mean) = [Σ SD^2^ + 2 Σ r_ij_ SD_i_ SD_j_]/9, applied to the three territorial standard deviations and their Pearson’s correlations. In both groups the observed dispersion of global longitudinal strain matches this prediction; so, the narrow control distribution is fully explained by the near-zero correlation between views in that group and requires no additional source of variance reduction. The larger value expected under independence is shown for comparison.

## Data Availability

The data presented in this study are not publicly available due to patient confidentiality and privacy restrictions. If permitted by legal regulations, anonymized data may be made available to qualified researchers upon request without personal identifiers.

## Abbreviations

2D	two-dimensional
A2C	apical two-chamber (view)
A3C	apical three-chamber (view)
A4C	apical four-chamber (view)
aCMQ	Philips speckle-tracking strain analysis software used for myocardial deformation measurements
AUC	area under the curve
AVC	automated speckle-tracking technique
BMI	body mass index
CASDTC	Child and Adolescent Substance Dependence Treatment Center
CG	control group
CI	confidence interval
DSM-5	Diagnostic and Statistical Manual of Mental Disorders, Fifth Edition
EDV	end-diastolic volume
ESV	end-systolic volume
FDR	false discovery rate
FE	Fisher’s exact test
GLS	global longitudinal strain
LV	left ventricle (ventricular)
LV EF	left ventricular ejection fraction
LVIDd	left ventricular internal diameter at end-diastole
LVIDs	left ventricular internal diameter at end-systole
LVPWd	left ventricular posterior wall thickness at end-diastole
MWU	Mann–Whitney U test
n	number of participants
NPV	negative predictive value
OR	odds ratio
PG	patient group
PPV	positive predictive value
Q1, Q3	first and third quartiles
R^2^	coefficient of determination (McFadden pseudo R^2^)
ROC	receiver operating characteristic
RVIDd	right ventricular internal diameter at end-diastole
